# Diet Quality and Weight Status are Predicted by Federal Nutrition Assistance Program Participation, Health, and Demographics

**DOI:** 10.1016/j.cdnut.2025.107505

**Published:** 2025-07-09

**Authors:** Lauren E Au, Charles D Arnold, Christabel Domfe, Lorrene D Ritchie, Shannon E Whaley, Marianne Bitler, Edward A Frongillo

**Affiliations:** 1 Department of Nutrition, University of California, Davis, Davis, CA, United States; 2 Division of Agriculture and Natural Resources, Nutrition Policy Institute, University of California, Oakland, CA, United States; 3 Public Health Foundation Enterprises Women, Infants, and Children, Irwindale, CA, United States; 4 Department of Economics, University of California, Davis, Davis, CA, United States; 5 National Bureau of Economic Research; 6 Department of Health Promotion, Education, and Behavior, University of South Carolina, Columbia, SC, United States

**Keywords:** HEI-2020, classification and regression tree, BMI*z*, diet, children, WIC, SNAP, Medicaid, race, ethnicity

## Abstract

**Background:**

Programs, such as Medicaid, the Supplemental Nutrition Assistance Program, and the Special Supplemental Nutrition Program for Women, Infants, and Children (WIC), provide access to vital medical and nutrition services. Few studies have investigated whether demographic, social, and economic determinants of health, including length of time spent on these programs, are associated with diet quality and weight status in early childhood.

**Objectives:**

Classification and regression tree analysis, a machine learning method, was used to determine health predictors to identify patterns of children with higher compared with lower diet quality and higher compared with lower weight status.

**Methods:**

Using the WIC infant and toddler feeding practices study-2 (unweighted *N* = 3051; weighted *N* = 413,211), classification and regression tree identified the sequence of binary splits that best differentiated the sample on Healthy Eating Index-2020 (HEI-2020; range 0–100) and HEI-2020 subscales (adequacy and moderation), and body mass index (BMI in kg/m^2^) *z*-score at 2–5 y. Predictors, including maternal BMI, child birthweight, sociodemographics, and length of time spent on safety net programs, were considered.

**Results:**

Higher HEI-2020 scores were primarily predicted by race and ethnicity (e.g., Hispanic, Spanish, or non-Hispanic White), and longer WIC and shorter Supplemental Nutrition Assistance Program duration. In examining HEI-2020 subscales, higher HEI adequacy was primarily predicted by higher education, older maternal age, longer WIC duration, and race and ethnicity. Higher HEI moderation was primarily predicted by race and ethnicity and longer WIC duration. Higher BMI *z*-score was primarily predicted by higher birth weight.

**Conclusions:**

Child diet quality and weight status were associated with different social determinants of health, which included maternal weight status, race and ethnicity, and food assistance program participation, particularly WIC.

## Introduction

Socioeconomic disparities in nutrition during childhood can contribute to poor diet quality and weight status [[1], [2], [3]]. Households with higher socioeconomic status (SES) are more likely to have lower food insecurity, healthier food habits, and more frequently meet national dietary guidelines compared to people with low SES; differences which can impact overall health [1,4,5]. Some families from low SES households have to choose between paying for housing, utilities, food, or medicine [6]. To help meet basic needs, social safety net programs, such as Medicaid, the Supplemental Nutrition Assistance Program (SNAP), and the Special Supplemental Nutrition Program for Women, Infants, and Children (WIC), provide children from low-income households access to vital medical and nutrition services for health and well-being. Historically, these safety net programs were created to provide health coverage and alleviate food insecurity and hunger for low-income people. Given the dramatic rise in childhood obesity over the past several decades [7], there has been an increased need for these programs to promote healthy dietary quality and weight status in low-income children [[8], [9], [10]].

Medicaid is the nation’s largest source of health coverage for low-income children [11]. Medicaid insures close to 39 million children [[11], [12], [13]]. Evidence shows that Medicaid coverage in early childhood puts individuals on a better health trajectory for long-term health [14,15]. Further, Medicaid coverage for adults has been associated with higher diet quality, lower obesity rates, improved access to care, and reduced mortality [16,17].

SNAP and WIC, 2 of the largest federal nutrition assistance programs, aim to improve nutrition and food security. SNAP provides households with an electronic benefit transfer card they can use to purchase eligible foods for approximately 42 million people, of which 36% are households with children [18]. WIC provides participating individuals a prescribed nutritious food package, nutrition education, and referrals to health and social services [19]. WIC serves >6 million pregnant and postpartum females and children aged ≤5 y per year [20]. Although WIC benefits are not intended to provide supplemental food for entire families, but only for pregnant females and children aged <5 y, when participants are also enrolled in SNAP, the effects of these 2 programs could be jointly beneficial. Prior research shows that participants who stay on SNAP tend to be more food insecure and have poorer diet quality than income-eligible nonparticipants [21]. In contrast, WIC participants tend to have better diet quality the longer they stay on the program, potentially due to the healthy food packages combined with nutrition education [22].

Many social determinants of health, including program participation, are related to health behaviors and health. Examining these predictors in relation to health behaviors and health cannot be modeled or understood easily using traditional regression methods. Classification and regression tree (CART) analysis is a powerful data-driven, machine learning method to identify prediction models without making parametric or distributional assumptions. CART builds trees through a sequence of binary splits that can best sort the sample on outcomes of interest. CART analysis has been used in studies related to child nutrition, such as examining childhood overweight and obesity [[23], [24], [25], [26]], childhood diet quality [27,28], risk factors for low and high birthweights [29,30], and breastmilk sodium concentration and weight loss in breastfeeding infants [31].

CART has also been utilized to investigate the effects of social safety net programs. For instance, it has been employed to identify predictors of WIC benefit usage behavior [32] and to determine factors associated with inpatient visits among Medicaid-insured patients [33]. CART analysis has not yet been used to investigate determinants of health; however, it includes the joint participation in WIC, SNAP, and Medicaid, and their association with child diet quality and weight status in early childhood. Thus, the objective of the current study was to apply CART analysis to identify the demographic, social, and economic determinants of health, including duration in specific safety net programs, that distinguish children from low-income households with higher compared with lower diet quality and higher compared with lower weight status.

## Methods

### Participants

The WIC Infant and Toddler Feeding Practices Study-2 (WIC ITFPS-2) is a nationally representative, longitudinal study of caregivers (>16 y) and their children followed from around birth up to age 9 y [34]. WIC ITFPS-2 is designed to examine feeding practices, the associations between WIC services and those practices, and the health and nutrition behaviors of children receiving WIC [34]. In 2013, study caregivers were recruited in person from 80 WIC sites across 27 states and territories. WIC sites were selected using a stratified 2-stage sampling approach [35]. Inclusion criteria included enrolling in WIC for the first time for that pregnancy or infant, and the ability to complete interviews in either English or Spanish. Exclusion criteria included: the child being aged >2.5 mo at the time of recruitment; being an adolescent mother aged <16 y; being a mother in foster care at the time of enrollment; and being a foster parent enrolling a foster infant.

Participants received a prenatal interview and ≤16 postnatal interviews during the first 5 y (1 mo, 3 mo, 5 mo, 7 mo, 9 mo, 11 mo, 13 mo, 15 mo, 18 mo, 24 mo, 30 mo, 36 mo, 42 mo, 48 mo, 54 mo, and 60 mo). Interviews were conducted by telephone by trained interviewers in English or Spanish. Interview questions included sociodemographic information, breastfeeding, and other feeding practices, as well as participation in WIC, SNAP, and Medicaid. Additionally, a 24-h dietary recall was collected at each time, except at 30 mo, 42 mo, and 54 mo. The study followed children at ages 6 y and 9 y, but the current analysis focuses on publicly available data of the core and supplemental samples through 5 y. The national study was approved by the Westat Institutional Review Board and the United States Office of Management and Budget. The national study is registered at clinicaltrials.gov as NCT02031978.

### Dietary intake

The majority of interviews after the prenatal interview included a 24-h dietary recall, administered over the phone, using the USDA automated multiple-pass method [36]. During the automated multiple-pass method, a caregiver was asked to recall all their child’s dietary intake for the previous day, which could include the weekday or weekend. The caregiver was asked to report all foods, beverages, and dietary supplements for each eating event [37]. For daycare foods, the caregiver obtained any missing details about foods from a knowledgeable source (i.e., daycare provider). If they do not know the amounts, the amounts are estimated based on the USDA food and nutrient database for dietary studies, 5.0 [38].

### Healthy eating index-2020

Healthy Eating Index-2020 (HEI-2020) total scores were computed using the population ratio method [39]. The HEI-2020 has been revised to reflect the 2020–2025 Dietary Guidelines for Americans and is considered a valid tool for assessing diet quality among individuals aged ≥2 y [40]. The total HEI-2020 score at 2–5 y was a primary outcome variable. In addition, the HEI-2020 adequacy subscale (sum of scores for total fruits, whole fruits, total vegetables, greens and beans, whole grains, dairy, total protein foods, seafood and plant proteins, and fatty acids) and the HEI-2020 moderation subscale (sum of scores for refined grains, sodium, added sugars, and saturated fats) were used as secondary outcomes.

### BMI *z*-scores

As part of regular clinic visits, WIC sites use standardized protocols to directly measure outcomes for most enrolled children. For participants who have left WIC, the study attempted to collect weight and length information from the child’s health care provider. BMI *z*-scores (BMI*z*) were calculated at 2–5 y using the Centers for Disease Control and Prevention age- and sex-specific growth charts [41] and are used as a primary outcome.

### Predictor variables

To capture the extent of program participation, a variable for the duration of Medicaid, SNAP, and WIC participation was calculated at each time point (24 mo, 36 mo, 48 mo, and 60 mo) using all prior survey rounds where the question was asked (Supplemental Table 1). The percentage of interviews where a caregiver reported that the child was receiving each program was used to define the duration groups, as has been done in previous studies [[42], [43], [44]]. Duration of program participation was then categorized using CART analyses, as described in the statistical analysis section.

Demographic characteristics of the study child, including sex, birth weight, and race and ethnicity, were collected from the caregiver at the first postnatal interview. Race and ethnicity were used in part as a marker of potential social and structural determinants of health that were otherwise unmeasured and could not be controlled for. Other sociodemographic variables collected from the study mother included maternal age at child’s birth, maternal race and ethnicity, language preference, maternal BMI, gestational diabetes, maternal depression score at the 3-mo visit, and household size. Variables collected annually included maternal BMI, marital status, household size, household income, household food security, and maternal employment status. Demographic characteristics were included using the closest in time measurement to the outcome to maximize their predictive relevance to the outcome and account for proximal confounding. Conditional multiple imputation was conducted to incorporate information from individuals with partial data using a conservative count of 10 imputations.

### Statistical analysis

CART analysis was implemented using a minimum terminal node size of 10% of the sample with cross-validated complexity parameter selection that pruned trees by selecting the smallest tree within a 1 SE equivalence margin of the tree with the lowest coefficient of variation. This process identified a sequence of binary splits among the candidate variables that best partitioned the sample on differences in the outcomes (diet quality and BMI*z*) as measured by maximizing the between-nodes sum-of-squares. This process led to a tree with a root node (full sample) from which branches emerged, and derivative nodes at each point were developed, and subgroups split into terminal nodes [45].

For ages 2 y, 3 y, 4 y, and 5 y, a separate CART analysis was fit for each age and each outcome (diet quality and BMI*z*). CART analyses were conducted with all program participation variables (Medicaid, SNAP, and WIC) included at the same time to explore joint participation between variables. CART was preferred to linear regression because of the possibility of complex interactions even among the first predictors. Additionally, sensitivity analyses were done in which the program participation variables were included separately. Duration of program participation was categorized into:

• Always: reporting participation in the program in the current and all previous timepoints
• Sporadic: reporting participation and nonparticipation
• None: reporting nonparticipation at each timepoint

Analyses were repeated using these duration categories for Medicaid (always/sporadic compared with none), SNAP (always, sporadic, and none), and WIC (always compared with sporadic), selected based on proportion of sample in each category and distribution of outcomes across the categories (Supplemental Table 2).

In all analyses, outcome-specific survey weights were used to have the sample represent the population and compensate for both the unequal sampling probabilities and nonresponse. In addition, estimation incorporated balanced replicated weights as recommended by the WIC ITFPS-2 study data use guidelines [35] to account for the complex survey design. The available sample size of 3051 participants provides 80% power to detect weak to strong associations between program duration and outcomes analogous to a strength of correlation of 0.06 or larger with the full sample, 0.08 or larger within a half-sample split, and 0.11 or larger within a quarter-sample split. The data analysis was conducted using the rpart statistical package in R version 4.1.24 [46].

## Results

Most caregivers in the sample were aged between 20 y and 25 y, were Hispanic, spoke English, and had a high school education or more (Table 1). The average HEI-2020 score (out of 100 points) was 56.0–57.6 between 2 and 5 y. The mean child BMI*z* score at 2 y was 0.69, decreasing to 0.61 by 5 y. Most participants were on WIC at 2 y, and this decreased to largely sporadic participation at 4–5 y. SNAP participation was more intermittent, especially at older years (Supplemental Table 2). More participants were on Medicaid sporadically and always compared to never across all years.

**TABLE 1:** Characteristics for participants in the Special Supplemental Nutrition Program for Women, Infants and Children Infant Toddler Feeding Practices Study-2.

Characteristic^1^	Unweighted *N* = 3051	Weighted *N* = 413,211
**Maternal age at childbirth** (*n*, %)^2^		
16–19 y	355 (11.6)	49,442 (12.0)
20–25 y	1256 (41.2)	165,655 (40.1)
26 y or older	1440 (47.2)	198,115 (47.9)
**Maternal race** (*n*, %)		
Hispanic	1217 (39.9)	197,419 (47.8)
Non-Hispanic White	895 (29.3)	109,857 (26.6)
Non-Hispanic African American	769 (25.2)	82,432 (19.9)
Non-Hispanic other	170 (5.6)	23,504 (5.7)
**Language preference** (*n*, %)		
Spanish	569 (18.7)	85,435 (22.2)
English	2479 (81.3)	299,263 (77.8)
**Marital status at enrollment** (*n*, %)		
Married	910 (29.8)	137,991 (33.3)
Not married^3^	2141 (70.2)	275,220 (66.6)
**Maternal education level at enrollment** (*n*, %)		
None through grade 11	741 (24.4)	100,996 (24.5)
High school	1166 (38.3)	156,343 (37.9)
More than high school	1135 (37.3)	155,458 (37.7)
**Maternal employment status at 6 mo child age** (*n*, %)^4^		
Full-time	563 (21.3)	74,390 (19.9)
Part-time	530 (20.0)	70,465 (18.9)
Not working for pay	1551 (58.7)	228,846 (61.2)
**Maternal BMI at screening** (*n*, %)		
Normal or underweight	1359 (44.5)	188,500 (45.6)
Overweight	833 (27.3)	101,023 (24.4)
Obese	859 (28.2)	123,688 (29.9)
**Household size at 6 mo child age** (*n*, %)^4^		
2 people	241 (9.1)	32,059 (8.6)
3 people	722 (27.1)	100,618 (27.0)
4 people	712 (26.8)	104,964 (28.1)
5 people or more	985 (37.0)	135,287 (36.3)
**Household poverty level at enrollment** (*n*, %)^4^^,^^5^		
75% of the poverty guideline or below	1956 (64.1)	257,597 (62.3)
Above 75% but <130%	809 (26.5)	111,536 (27.0)
Above 130% of the poverty guideline	286 (9.4)	44,079 (10.7)
**Household food security at enrollment**		
Food secure	1986 (65.5)	272,571 (66.0)
Low food security	677 (22.3)	93,682 (22.7)
Very low food security	368 (12.1)	46,958 (11.4)
**Child sex, female** (*n*, %)	1495 (49.0)	198,129 (47.9)
**Child weight at birth (g)**		
Low (<2.5 kg)	222 (7.3)	32,341 (7.8)
Normal (2.5 kg to <4.5 kg)	2788 (91.4)	375,350 (90.9)
High (>4.5 kg)	41 (1.3)	5520 (1.3)
**BMI*z* score at 2 y** (mean, SD)	0.70 (1.27)	0.69 (1.28)
**BMI*z* score at 3 y** (mean, SD)	0.50 (1.32)	0.52 (1.29)
**BMI*z* score at 4 y** (mean, SD)	0.49 (1.39)	0.53 (1.38)
**BMI*z* score at 5 y** (mean, SD)	0.56 (1.30)	0.61 (1.28)
**HEI-2020 at 2 y** (mean, SD)	56.3 (11.1)	56.7 (10.9)
**HEI at 3 y** (mean, SD)	57.5 (12.1)	57.6 (12.1)
**HEI at 4 y** (mean, SD)	56.6 (12.5)	57.0 (12.7)
**HEI at 5 y** (mean, SD)	55.4 (12.7)	56.0 (12.6)

Abbreviations: BMI *z*-score, body mass index *z*-score; HEI-2020, Healthy Eating Index-2020; SD, standard deviation.

1 Values are means ± SDs or frequency (percentage)

2 Maternal includes other primary caregivers if not the mother (<1% of respondents are caregivers other than the infant’s biological mother at the time of enrollment).

3 Not married includes divorced, widowed, or separated.

4 Because of missing values, the total *n* is not the same for all variables.

5 Income at 100% of the federal poverty level was $23,550 for a family of 4 in 2013.

At 2 y, 3 y, and 5 y old, race and ethnicity were the first predictors to distinguish total HEI-2020 scores (Table 2). At 3 y, the second and third predictors fluctuated between WIC and SNAP duration, maternal education, race and ethnicity, and income. At 4 y old, WIC duration was the first predictor of HEI-2020, then SNAP duration and race and ethnicity.

**TABLE 2:**
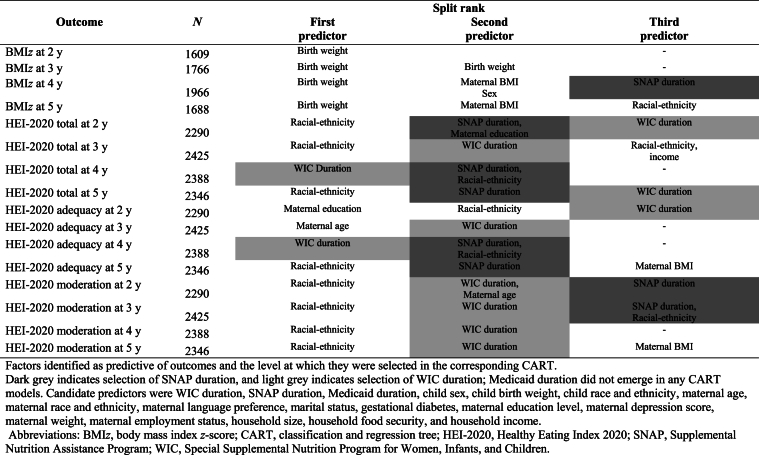
Predictors of BMI *z*-score and Healthy Eating Index-2020 outcomes by classification and regression tree analysis for children in the Special Supplemental Nutrition Program for Women, Infants and Children Infant Toddler Feeding Practices Study-2.

In examining HEI-2020 subscales, at 2 y, HEI adequacy was primarily predicted by maternal education, race and ethnicity, then WIC duration (Table 2). At 3 y, maternal age was the first predictor to distinguish HEI adequacy, followed by WIC duration. At 4 y, WIC duration, then SNAP duration, and race and ethnicity defined HEI adequacy. After race and ethnicity, SNAP duration, and then maternal BMI were important predictors of HEI adequacy at 5 y. Race and ethnicity, then WIC duration, were the first 2 predictors to distinguish HEI moderation across 2–5 y. SNAP duration was the third most important predictor at ages 2 and 3 for HEI moderation.

Birth weight was consistently the most important predictor distinguishing BMI*z* across 2–5 y (Table 2). After birth weight, maternal BMI, gender of the child, and SNAP duration were important predictors of BMI*z* at 4 y old. At 5 y, maternal BMI was the second and race and ethnicity were the third predictors to distinguish BMI*z*.

**FIGURE 1:**
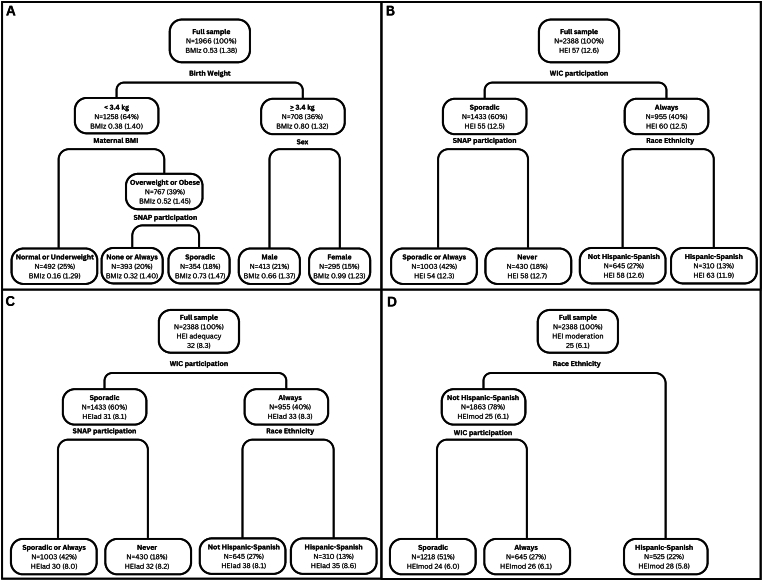
Classification trees for 4-y-old outcomes in the Special Supplemental Nutrition Program for Women, Infants and Children (WIC) Infant Toddler Feeding Practices Study-2: (A) BMI *z*-scores (BMI*z*); (B) Healthy Eating Index-2020 (HEI) scores; (C) HEI adequacy (HEIad) scores; and (D) HEI moderation (HEImod) scores. BMI, body mass index; SNAP, Supplemental Nutrition Assistance Program.

Although there was variation in selected predictors across child ages, the directions of the associations were generally consistent within an outcome, as illustrated in Figure 1 and Supplemental Figures 1–3. Specifically, for BMI*z* at 4 y (Figure 1A), higher birthweight (≥3.4 kg) was associated with a higher BMI*z*, which was then split by sex, with higher BMI*z* among female children. Among those with lower birthweight, having a mother who was normal weight or underweight was associated with lower BMI and among those with a mother who was overweight or obese, sporadically participating in SNAP corresponded to having a lower BMI*z*, whereas those always or never participating in SNAP having higher BMI*z* (0.32 BMI*z* score compared with 0.73 BMI*z* score). For HEI at 4 y (Figure 1B), those with sporadic participation in WIC had lower HEI as compared to those always participating in WIC (55 compared with 60). With no participation in SNAP, participants had even lower HEI as compared to those sporadically or always participating in SNAP (54 compared with 58). Among those who were always participating in WIC, being of Hispanic descent and predominantly Spanish speaking was associated with higher HEI compared with those who were not. The pattern of association was the same for the HEI adequacy subscale (Figure 1C), but not for the HEI moderation subscale (Figure 1D), which split first on race and ethnicity (with higher HEI moderation among Hispanic-Spanish speaking children) and then on WIC duration, with higher scores among those always on WIC.

## Discussion

Child diet quality was associated with several social determinants of health predictors, which included measures of health, race and ethnicity, and food assistance program participation, particularly WIC. The key predictor distinguishing weight status was birth weight or maternal BMI, whereas race and ethnicity, and WIC and SNAP duration were the dominant predictors of total HEI-2020 scores. Race and ethnicity and longer WIC duration were the primary predictors of higher adherence scores on the HEI subscale for moderation, which reflects how much a person adheres to the foods to limit in the United States Dietary Guidelines for Americans, such as refined grains, sodium, added sugars, and saturated fats [40].

Diet quality was primarily predicted by race and ethnicity and, to a lesser extent, by the duration of participation in food assistance programs, particularly consistent participation in WIC. These findings align with previous research showing an association between race and ethnicity and diet quality [47], with higher diet quality among Spanish-speaking Hispanics compared to English-speaking Hispanics [48], and diets of Hispanic children having better nutrient densities and lower caloric densities than those of non-Hispanic (NH) Whites, whereas NH Black children had poorer nutrient densities [49]. Additionally, within the normal weight category, Mexican American children have been shown to have better diet quality than NH Black children [50]. The differences in diet quality observed within and across racial and ethnic groups can be partially attributed to whether the primary caregiver was born in the United States [47], the degree of acculturation [48,51], and the child’s BMI*z* categorization [50]. Additionally, there has been a documented association between lower SES and poorer diet quality, with a growing trend observed among Black children [52] – a pattern that was also evident in this study population.

Although the mean HEI scores for children aged 2 through 5 in this study population were consistently lower than the HEI-2020 and HEI-2015 United States population average of 61 out of 100 for ages 2 through 4 [53,54], which is consistent with previous research [50,51], this study identified subpopulations of low-income children who may have better diet quality, specifically children whose caregivers were Hispanic-Spanish speaking and also participated in WIC longer. Notably, prior research has shown that being Hispanic-Spanish speaking is associated with higher odds of longer WIC participation, which in turn is associated with better diet quality in children [43]. It remains unclear why this subgroup of participants consistently has better diet quality, but it may be due to a greater appreciation of the WIC food package and/or the nutrition education provided for the full period of WIC eligibility to age 5. Given these findings, the possible importance of long-term WIC participation in improving diet quality cannot be downplayed, particularly for some racial and ethnic groups. By providing a nutritious food package, WIC may potentially reduce the intake of nutrients of concern, such as added sugar and sodium, while promoting the consumption of healthy food groups, such as fruits and whole grains, as highlighted in the Dietary Guidelines for Americans [53].

Findings in previous literature [55,56] have shown that children from households participating in SNAP had lower HEI scores at ages 2–5 y compared with income-eligible nonparticipants, suggesting poorer diet quality. This study observed a similar but more nuanced pattern. Specifically, sporadic or continuous participation in SNAP was associated with lower diet quality, but only among children with intermittent WIC participation or those who were NH White. In contrast, children who were never on SNAP but consistently participated in WIC or belonged to racial and ethnic groups other than NH White had higher diet quality. Notably, SNAP participants have been reported to experience higher food insecurity rates compared to nonparticipants, although this may not be causally attributed to SNAP use [[57], [58], [59], [60]]. Although individuals participating in 2 or more safety net programs are more likely to be food insecure [61], have lower incomes, and experience economic hardships [62], consistent joint participation in WIC may improve the diet quality of SNAP participants, as shown by our study findings. Regarding the sporadic participation trend that was observed for SNAP participants, previous research shows that half of all participants stop receiving benefits within 12 mo, and two-thirds leave within 2 y [63]. In addition, SNAP participants often experience income volatility and job turnover [63], affecting program eligibility, and the SNAP program has requirements for frequent recertification of eligibility, leading to sporadic participation, which potentially affects long-term impacts on child diet quality in these households and the assessment of those impacts itself. Finally, although previous studies of adults [16,17] have indicated an association between Medicaid coverage and better diet quality, this study found that the duration of Medicaid did not significantly impact child diet quality. This may be partly because this study asked about household Medicaid and household SNAP participation rather than direct participation by the child.

CART analysis identified birth weight as the primary predictor for early childhood weight status, which aligns with previous studies that observed higher BMI*z* scores in children with increased birth weights [64,65] and accelerated weight gain patterns [66]. Additionally, in a similar cohort that was predominantly from low-income and multiethnic households, increased birth weight was independently predictive of higher childhood obesity rates [67]. Other studies that included birth weight in their models also identified it as a strong predictor of childhood obesity [68,69]. In addition to birth weight, increased maternal BMI was associated with higher BMI*z* scores [65], doubling the risk of obesity at 2–4 y among low-income children enrolled in WIC [70], consistent with this study’s findings.

Strengths of this study include the use of a national, longitudinal cohort with information about Medicaid, SNAP, and WIC self-reported participation collected at 8 different time points through the first 5 y of life. In addition, BMI*z* and HEI-2020 scores, which require time-intensive data collection, were available at 2–5 y of age, which are not available in many longitudinal data sets that include safety net program duration. Because participants in this study were predominantly WIC participants recruited from WIC sites, their SNAP and Medicaid participation may be systematically different and influenced by self-selection bias, potentially limiting the representativeness of households participating in Medicaid and SNAP within this population of low-income households. Because Medicaid, SNAP, and WIC participation were not assessed between study visits, it is possible that the duration groups do not accurately reflect sporadic program participation or churning, a topic worth further research. Further, because self-reports of program participation may be underreported, there may have been a misclassification of some people who participate less extensively as nonparticipants [71]. Further, there might be a selection as to who stays on the programs or leaves them. Such selection may lead to systematic differences in the estimates of the effects of some programs. Despite this limitation, the extensive longitudinal data collection of WIC ITFPS-2 makes it well-suited to use CART analysis to investigate the social determinants of health, including duration in specific safety net programs, that distinguish children from low-income households with higher compared with lower diet quality and higher compared with lower weight status. Additionally, a single day of 24-h dietary recall was used to calculate HEI-2020 scores, which may limit the ability to assess usual intake and may contribute to potential energy underreporting [72].

In conclusion, child diet quality and weight status were associated with several social determinants of health and often occurred at the intersection of multiple predictors, which included maternal BMI, race and ethnicity, and food assistance program duration of participation, particularly WIC. The CART categorization of social safety net programs into ordinal categories provides a framework for examining needs specific to the duration of participation. These findings can help inform tailored recommendations, such as staying on the WIC program for longer, for improving diet quality and weight status of young children.

## Author contributions

The authors’ responsibilities were as follows – LEA: conceptualized, designed the study, drafted the manuscript, and revised the manuscript; CDA: designed the study, carried out the analyses, critically reviewed, and revised the manuscript; CD: assisted in the investigation, helped to draft the manuscript, and revised the manuscript; LDR, SEW, MB: designed the study, critically reviewed, and revised the manuscript; EAF: designed the study, critically reviewed, and revised the manuscript; and all authors: read and approved the final manuscript.

## Data availability

Data described in the manuscript and a code book are publicly and freely available without restriction at: https://data.nal.usda.gov/dataset/wic-infant-and-toddler-feeding-practices-study-2-wic-itfps-2-prenatal-infant-year-5-year-datasets and https://osf.io/suahz/.

## Funding

This publication was supported by grant #81359 from Healthy Eating Research, a national program of the Robert Wood Johnson Foundation and the USDA/National Institute of Food and Agriculture Hatch Project# CA-D-NTR-2689-H. The content is solely the responsibility of the authors and does not necessarily represent the official views of the Robert Wood Johnson Foundation or the USDA.

## Conflict of interest

The authors report no conflicts of interest.
