# Supplementary material for: Diet Quality and Weight Status are Predicted by Federal Nutrition Assistance Program Participation, Health, and Demographics
Source: Curr Dev Nutr. 2025 Jul 9;9(8):107505. doi: 10.1016/j.cdnut.2025.107505 (PMC12320653; doi:10.1016/j.cdnut.2025.107505)
Supplement: multimedia component 1 [file mmc1.docx]

| **Supplemental Table 1**. Prevalence of program participation by survey round in the in the Special Supplemental Nutrition Program for Women, Infants and Children (WIC) Infant Toddler Feeding Practices Study-2. | | | | | | |
| --- | --- | --- | --- | --- | --- | --- |
|  | **WIC participation** | | **Supplemental Nutrition Assistance Program (SNAP) participation**^a^ | | **Medicaid participation**^a^ | |
| **Age at Survey** | **N** | **%** | **N** | **%** | **N** | **%** |
| Enrollment | 3051 | 100% | 3038 | 49.6% | 3015 | 75.7% |
| Month 1 | 2753 | 96.6% |  |  |  |  |
| Month 3 | 2342 | 97.4% |  |  |  |  |
| Month 5 | 2167 | 96.8% |  |  |  |  |
| Month 7 | 2654 | 95.9% |  |  |  |  |
| Month 9 | 2086 | 95.3% |  |  |  |  |
| Month 11 | 2003 | 94.1% |  |  |  |  |
| Month 13 | 2460 | 86.4% | 2453 | 52.8% | 2433 | 74.8% |
| Month 15 | 1830 | 81.4% |  |  |  |  |
| Month 18 | 1771 | 78.0% |  |  |  |  |
| Month 24 | 2296 | 68.6% | 2285 | 50.3% | 2283 | 75.3% |
| Month 30 | 2413 | 60.5% | 2412 | 50.9% | 2408 | 74.7% |
| Month 36 | 2423 | 56.6% | 2421 | 48.7% | 2413 | 73.1% |
| Month 42 | 2441 | 53.9% | 2441 | 48.7% | 2434 | 76.3% |
| Month 48 | 2386 | 50.9% | 2386 | 46.0% | 2380 | 72.9% |
| Month 54 | 2362 | 49.3% | 2362 | 45.9% | 2357 | 73.6% |
| Month 60 | 2350 | 44.0% | 2343 | 42.7% | 2337 | 71.5% |
| ^a^SNAP and Medicaid household participation was not collected at 1, 3, 5, 7, 9, 11, 15, or 18 months. | | | | | | |

Manuscript ID: CDN-D-24-00436R1
Article Title: Diet quality and weight status is predicted by federal nutrition assistance program participation, health, and demographics

First Author: Lauren Au

| **Supplemental Table 2** Study outcomes by program participation and age of the child in the Special Supplemental Nutrition Program for Women, Infants and Children (WIC) Infant Toddler Feeding Practices Study-2. | | | | | | | | |
| --- | --- | --- | --- | --- | --- | --- | --- | --- |
|  |  | **WIC** | | **Supplemental Nutrition Assistance Program (SNAP)** | | | **Medicaid** | |
|  |  | **Sporadic** | **Always** | **None** | **Sporadic** | **Always** | **None** | **Sporadic/Always** |
| **2 y** | **N (%)** | 1001 (33.0) | 2030 (67.0) | 1046 (34.3) | 943 (30.9) | 1058 (34.7) | 376 (12.4) | 2675 (87.7) |
| **BMIz** | **Mean (SD)** | 0.45 (1.33) | 0.77 (1.25) | 0.67 (1.22) | 0.64 (1.37) | 0.78 (1.23) | 0.59 (1.30) | 0.71 (1.27) |
| **HEI** | **Mean (SD)** | 55.13 (10.95) | 57.11 (11.13) | 57.72 (10.84) | 55.93 (10.96) | 55.56 (11.39) | 56.99 (10.07) | 56.31 (11.22) |
|  | | | | | | | | |
| **3 y** | **N (%)** | 1510 (49.9) | 1521 (50.2) | 925 (30.3) | 1255 (41.2) | 868 (28.5) | 306 (10.1) | 2745 (89.9) |
| **BMIz** | **Mean (SD)** | 0.46 (1.32) | 0.53 (1.32) | 0.51 (1.29) | 0.53 (1.36) | 0.46 (1.28) | 0.41 (1.31) | 0.51 (1.32) |
| **HEI** | **Mean (SD)** | 55.77 (11.73) | 59.39 (12.25) | 58.56 (11.92) | 57.51 (12.42) | 56.18 (11.69) | 56.97 (11.85) | 57.51 (12.14) |
|  | | | | | | | | |
| **4 y** | **N (%)** | 1768 (58.4) | 1264 (41.7) | 848 (27.8) | 1446 (47.4) | 754 (24.7) | 269 (8.8) | 2782 (91.2) |
| **BMIz** | **Mean (SD)** | 0.46 (1.36) | 0.51 (1.42) | 0.46 (1.36) | 0.54 (1.42) | 0.41 (1.34) | 0.30 (1.48) | 0.50 (1.38) |
| **HEI** | **Mean (SD)** | 55.15 (12.23) | 59.01 (12.58) | 58.41 (12.45) | 55.90 (12.23) | 56.03 (12.95) | 57.26 (12.96) | 56.54 (12.46) |
|  | | | | | | | | |
| **5 y** | **N (%)** | 1966 (64.9) | 1066 (35.2) | 794 (26.0) | 1595 (52.3) | 660 (21.6) | 248 (8.1) | 2803(91.9) |
| **BMIz** | **Mean (SD)** | 0.51 (1.27) | 0.67 (1.36) | 0.53 (1.27) | 0.61 (1.28) | 0.47 (1.40) | 0.39 (1.23) | 0.57 (1.30) |
| **HEI** | **Mean (SD)** | 54.41 (12.32) | 57.75 (13.16) | 56.66 (12.99) | 55.30 (12.58) | 53.98 (12.31) | 55.43 (12.36) | 55.38 (12.68) |
| Footnote: Body mass index z-score (BMIz), Healthy Eating Index-2020 score (HEI). | | | | | | | | |

**Supplemental Figure 1**. Classification trees for 2-year-old outcomes in the Special Supplemental Nutrition Program for Women, Infants and Children (WIC) Infant Toddler Feeding Practices Study-2: (A) body mass index z-scores (BMIz); (B) Healthy Eating Index-2020 (HEI) scores; (C) HEI adequacy scores; and (D) HEI moderation scores.


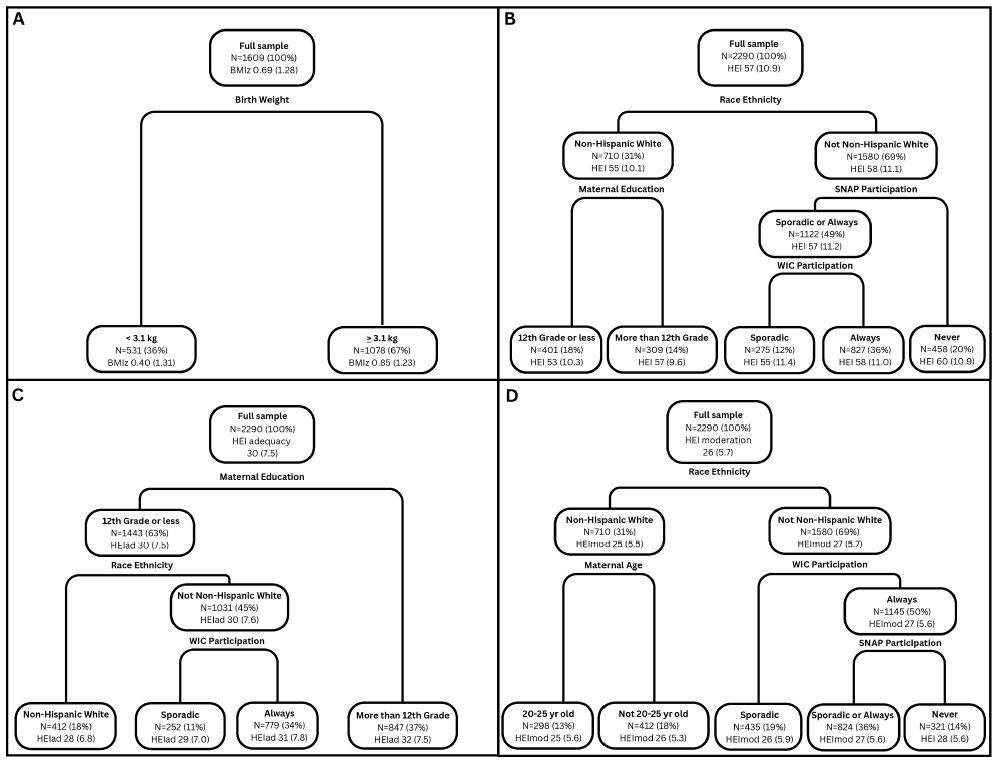


Footnote: Special Supplemental Nutrition Program for Women, Infants and Children (WIC), Supplemental Nutrition Assistance Program (SNAP), body mass index z-scores (BMIz), Healthy Eating Index-2020 score (HEI), HEI adequacy scores (HEIad), and HEI moderation scores (HEImod).

**Supplemental Figure 2**. Classification trees for 3-year-old outcomes in the Special Supplemental Nutrition Program for Women, Infants and Children (WIC) Infant Toddler Feeding Practices Study-2: (A) body mass index z-scores (BMIz); (B) Healthy Eating Index-2020 (HEI) scores; (C) HEI adequacy scores; and (D) HEI moderation scores.


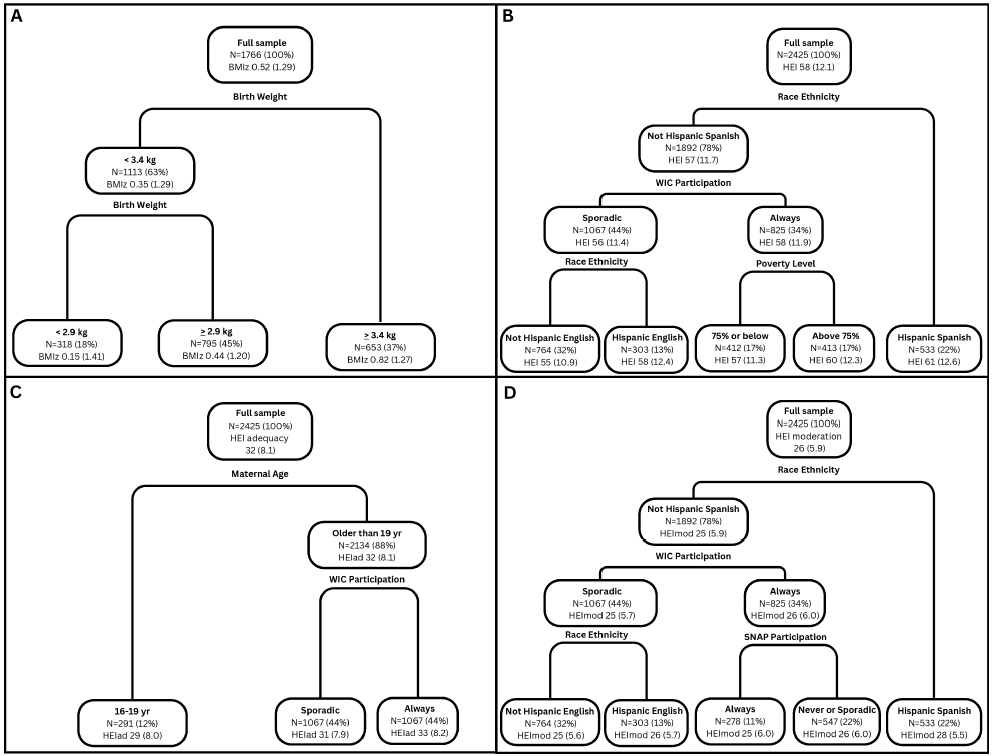


Footnote: Special Supplemental Nutrition Program for Women, Infants and Children (WIC), Supplemental Nutrition Assistance Program (SNAP), body mass index z-scores (BMIz), Healthy Eating Index-2020 score (HEI), HEI adequacy scores (HEIad), and HEI moderation scores (HEImod).

**Supplemental Figure 3**. Classification trees for 5-year-old outcomes in the Special Supplemental Nutrition Program for Women, Infants and Children (WIC) Infant Toddler Feeding Practices Study-2: (A) body mass index z-scores (BMIz); (B) Healthy Eating Index-2020 (HEI) scores; (C) HEI adequacy scores; and (D) HEI moderation scores.


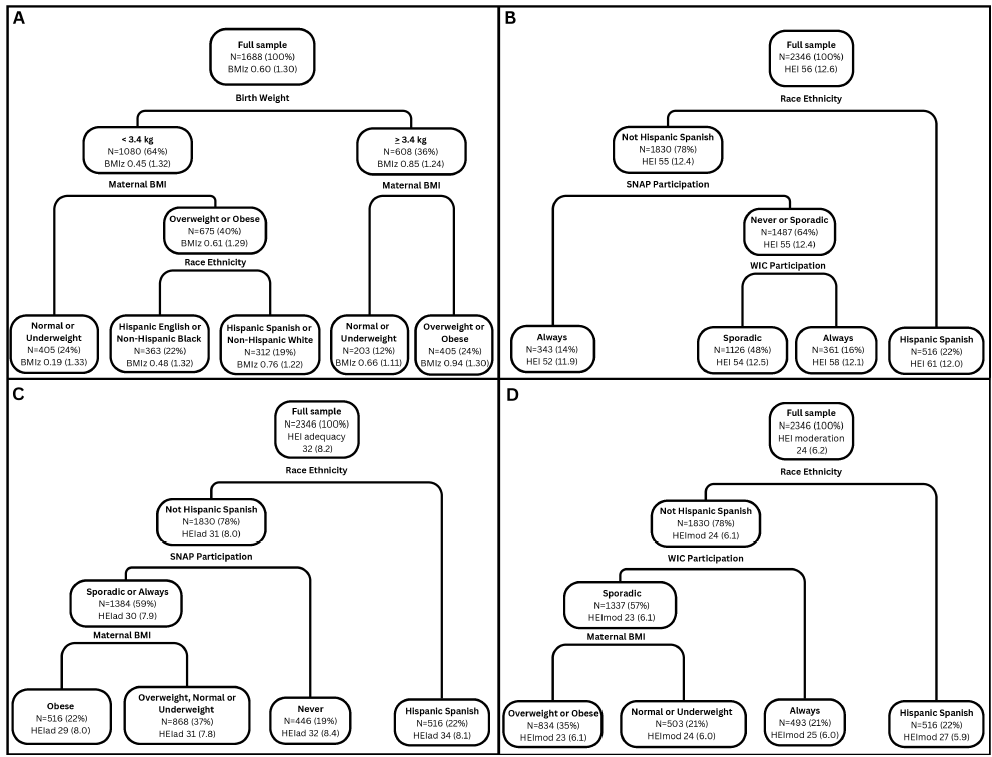


Footnote: Special Supplemental Nutrition Program for Women, Infants and Children (WIC), Supplemental Nutrition Assistance Program (SNAP), body mass index z-scores (BMIz), Healthy Eating Index-2020 score (HEI), HEI adequacy scores (HEIad), and HEI moderation scores (HEImod).
